# Retraction: microRNA-98 inhibits the proliferation, invasion, migration and promotes apoptosis of breast cancer cells by binding to HMGA2

**DOI:** 10.1042/BSR-2018-0571_RET

**Published:** 2021-06-08

**Keywords:** Breast cancer, High mobility group AT-hook 2, Invasion, microRNA-98, Proliferation

This Retraction follows an Expression of Concern relating to this article previously published by Portland Press.

This article is being retracted from *Bioscience Reports* at the request of the Editor-in-Chief and the Editorial Board following receipt of a notification from a reader, alerting the Editorial Board to the Western blots in Figure 4B which are duplicated in several other publications, as well as duplications in Figures 7A and 7C.

The authors have been contacted in regards to the retraction, and have not responded to the Journal's queries and the concerns raised. Given the extent of the issues raised, the Editorial Board stand by the decision to retract the article.

